# Open-Source R Shiny Dashboard (Global Burden of Disease Analysis Suite) for Multimethod Burden of Disease Research: Development and Evaluation Study

**DOI:** 10.2196/98718

**Published:** 2026-09-11

**Authors:** Belal Mohamed Hamed, Mohammed Tarek Hasan, Omar Ahmed Abdelwahab, Moaz Elsayed Abouelmagd, Ramez M Odat, Yasar Sattar, Ramy Mohamed Ghazy

**Affiliations:** 1 Faculty of Medicine Al-Azhar University Cairo Egypt; 2 Al-Mabara Health Insurance Hospital Zagazig, Sharqia Egypt; 3 Kasr-Alainy Faculty of Medicine Cairo University Cairo Egypt; 4 Faculty of Medicine Jordan University of Science and Technology Irbid Jordan; 5 Department of Interventional Cardiology West Virginia University Morgantown, WV United States; 6 High Institute of Public Health Tropical Health Department Alexandria University Alexandria Egypt; 7 College of Medicine King Khalid University Abha Saudi Arabia

**Keywords:** global burden of disease, R Shiny, web application, dashboard software, open-source software, reproducible research, data visualization, epidemiology, EAPC, estimated annual percentage change, joinpoint regression, time-series forecasting, health inequality, decomposition

## Abstract

**Background:**

The Global Burden of Disease (GBD) study provides the most comprehensive global burden estimates available, but translating GBD data downloads into structured analyses requires an integrated, multimethod workflow spanning trend estimation, changepoint detection, demographic decomposition, frontier benchmarking, and health inequality measurement. Existing tools cover portions of this workflow, but none integrate it end-to-end in a code-free interface, creating a substantial technical barrier, particularly for investigators in resource-limited settings.

**Objective:**

This study aimed to develop and release the GBD Analysis Suite, an open-source R Shiny dashboard that consolidates 11 commonly used analytical modules for GBD-style burden-of-disease research into a single, code-free, web interface supporting user-uploaded data, and to describe its underlying architecture, analytical methodology, and intended applications.

**Methods:**

The application was built using R 4.5.3 and the Shiny framework, integrating established statistical packages, including *forecast*, *segmented*, *openxlsx*, *ggplot2*, *plotly*, and others, into modules covering data import, choropleth mapping, estimated annual percentage change, joinpoint regression, Kitagawa and Das Gupta decomposition, multimodel time-series forecasting, descriptive visualization, sociodemographic index–based frontier analysis, descriptive age-period-cohort visualization, slope and relative indices of inequality, and batch export. The application accepts Global Health Data Exchange (GHDx)–formatted CSV or Excel files, automatically detects standard variable names, and enforces internal consistency through built-in validation checks. Kitagawa decomposition uses the symmetric mid-point formulation to ensure exact additivity. The three ensemble forecast models (autoregressive integrated moving average [ARIMA], exponential smoothing state-space [ETS], and a feed-forward neural network [NNETAR]) apply Box-Cox log transformation, with an explicit nonnegativity floor applied to the point forecast and prediction intervals of every model.

**Results:**

The application is publicly deployed and can be accessed through any modern web browser without requiring local installation. On the bundled 196-country, 34-year demonstration dataset, exporting the full set of outputs completes in approximately 12 seconds at a peak memory footprint of approximately 600 MB. A feature comparison against 5 existing tools (GBD Compare, GBD Foresight, NCI Joinpoint, BAPC, and NordPred) across 14 capability dimensions confirms that the GBD Analysis Suite is the only reviewed platform combining user-uploaded data input with code-free access to all 6 core analytical methods of modern GBD papers. Source code is publicly available under the MIT license.

**Conclusions:**

The GBD Analysis Suite lowers the technical barrier to reproducible, multimethod burden of disease analyses. It is designed particularly for researchers in low- and middle-income countries where biostatistical support, commercial software licenses, and high-performance computing are often unavailable. The modular architecture supports flexibility across disease areas, geographies, and time windows, subject to input data compatibility. Community contributions, feature requests, and collaborative extensions are welcomed through the project’s public GitHub repository.

## Introduction

The Global Burden of Disease (GBD) study, coordinated by the Institute for Health Metrics and Evaluation (IHME), provides the most comprehensive global estimates of disease incidence, mortality, and disability-adjusted life-years available, covering 204 countries and territories from 1990 to the present [1]. Its public data portal, the Global Health Data Exchange (GHDx), hosts extensive standardized datasets freely downloadable in CSV format [2]. Translating these downloads into export-formatted outputs (manuscript tables, formatted figures, and reproducible methods text) requires a multimethod workflow spanning at least 6 analytical domains: temporal trend analysis (estimated annual percentage change [EAPC]), changepoint detection (joinpoint regression), demographic decomposition, multimodel forecasting, frontier benchmarking, and health inequality measurement. Executing these methods reproducibly within a single project typically requires integrating multiple software packages, each with separate installation, documentation, and output formats, a substantial technical barrier for research teams with limited computational resources.

Existing tools address parts of this workflow, but none integrates the complete pipeline. Visualization portals such as IHME’s GBD Compare [3] and its companion GBD Foresight tool [4] enable exploration of precomputed estimates but provide no reanalysis capability. Analytical packages, including the National Cancer Institute (NCI) Joinpoint Regression Program [5], the R *forecast* package [6], and Bayesian age-period-cohort modeling tools such as BAPC [7] and NordPred [8], each address a single method and, in most cases, require local installation and programming fluency. No currently available free tool integrates EAPC estimation, joinpoint regression, decomposition, multimodel forecasting, frontier analysis, and inequality measurement in a single code-free interface. Previously published open-source R Shiny applications in epidemiology have typically targeted a single disease area or analytical task, such as cancer-registry analytics [9] or spatial and spatio-temporal disease mapping [10], rather than the integrated multimethod GBD workflow.

This fragmentation creates a substantial and disproportionate barrier for investigators in low- and middle-income countries (LMICs), where GBD data are most urgently needed to inform health policy, but biostatistical expertise, institutional computing infrastructure, and commercial software licenses are least available. Documented structural barriers to research capacity and analytical participation for investigators based in LMICs compound this gap [11]. For such investigators, a browser-based, user-uploadable, multimethod analytical platform would substantially lower the barrier between data availability and peer-reviewed publication.

This paper describes the GBD Analysis Suite: an open-source R Shiny dashboard implementing 11 commonly used analytical modules that together support the complete workflow of a GBD burden of disease study. The application requires no programming skills beyond data upload, runs in any modern web browser, and is freely deployable to shinyapps.io without institutional infrastructure. We describe its software architecture, data pipeline, analytical modules, output formats, and intended use cases, and compare its capabilities against 5 established tools covering parts of the GBD analytical workflow.

## Methods

### Data Input Model

The application accepts any dataset downloaded from the GHDx [12] in CSV or Microsoft Excel (.xlsx) format. Users may upload a single file or combine multiple disease-specific GHDx exports. A companion sociodemographic index (SDI) file, also available from the GHDx portal, may be uploaded separately to activate SDI-dependent modules (EAPC versus SDI correlation, frontier analysis, and global inequality).

The application’s automatic column-detection algorithm identifies standard GHDx variable names case-insensitively (location name, year, value, upper, lower, measure name, metric name, sex name, age name, and cause name), requiring only that location name, year, and value be present for core modules to function. Additional variables unlock specific analyses automatically. For example, the presence of both number and rate metrics activates Kitagawa decomposition. The presence of age-stratified rows additionally activates Das Gupta 3-component decomposition. A preformatted SDI template is available for download from within the application.

### Software Architecture

The GBD Analysis Suite was developed in R version 4.5.3 [13] using the *shiny* [14] and *shinydashboard* [15] frameworks. The interface is organized as an 11-tab sidebar dashboard. Core dependencies include *dplyr* [16], *tidyr* [17], and *data.table* [18] for data manipulation, *ggplot2* [19] and *plotly* [20] for visualization, *forecast* [6] for time-series modeling, *segmented* [21] for joinpoint regression, *maps* [22] for world polygon layers, *openxlsx* [23] for Excel export, and *officer* [24] and *flextable* [25] for Word-compatible methods-text generation. Complete version information for all packages is provided in Multimedia Appendix 1. All interactive charts are rendered via *plotly*, ensuring cross-browser compatibility without client-side R installation. The application is deployed as a Shiny app [26] and is fully operational when run locally via shiny::runApp(). Source code, bundled demonstration data, and a package-installation script are publicly available at the GitHub repository [27].

### Analytical Modules

#### Module 1: Data Import and Validation

Users can upload any GHDx-formatted CSV or Excel file via a browser-based file picker or load a bundled demonstration dataset with a single click. The demonstration dataset contains GBD 2023 meningitis estimates for incidence and mortality, reported as numbers, rates, and percentages, for both sexes combined and for males and females separately, across four age bands together with age-standardized estimates, spanning 1990 to 2023 across 196 countries and territories (599,760 rows). The dataset forms a complete factorial grid and contains no missing values by design. A small number of observations (1277 of 599,760) carry a value of exactly zero. These are retained on import and are excluded only by the positivity filter applied in the modules that require logarithmic transformation. Upon upload, the application automatically detects column mappings, verifies required fields, aggregates observations by measure, metric, sex, and age group, and generates a structured data-quality report.

#### Module 2: Choropleth Maps

Age-standardized rates are plotted as interactive choropleth world maps using *maps* package polygons joined to GHDx location names by a standardized name-matching table. Three map types are available: single-year age-standardized rate (ASR), EAPC magnitude, and percentage change between two user-selected years. Color scales use the *viridis* sequential palette for rate magnitude and percentage change displays, and the *RColorBrewer* RdYlGn diverging palette for EAPC magnitude, with user-adjustable breakpoints. Location names that do not match the bundled polygon set are left unfilled (rendered in neutral grey) without interrupting rendering, and subnational entries are not aggregated to the national level. Users should therefore verify that uploaded location names follow GBD standard naming conventions.

#### Module 3: EAPC

For each location, a log-linear model is fitted to age-standardized rates over the selected year range: ln (ASR) = alpha + beta × year. The EAPC is computed as 100 × (exp(beta) – 1) and its 95% confidence interval as 100 × (exp (beta +/– 1.96 × SE) – 1), following the partitioning method commonly applied in international cancer and burden of disease statistics [28]. The regression is fitted to the point estimate of the age-standardized rate only. Where a dataset supplies upper and lower uncertainty bounds, these columns are detected and retained but are not propagated into the EAPC estimate or its confidence interval, so the reported interval reflects sampling variability around the fitted log-linear trend rather than the underlying uncertainty of the GBD estimates themselves. Trend classification follows established conventions: a statistically significant positive EAPC (ie, confidence interval lower bound >0) is classified as increasing, while a statistically significant negative EAPC (ie, confidence interval upper bound <0) is classified as decreasing. Otherwise, the trend is classified as stable. This increasing/decreasing/stable classification assumes approximately independent annual observations. In the presence of strong temporal autocorrelation, it should be corroborated with methods that explicitly model serial dependence, such as joinpoint regression. Results are displayed as a sortable data table, a forest plot, a scatter of EAPC against baseline ASR, and, when an SDI file is provided, a scatter of EAPC against SDI with a loess smoothing line.

#### Module 4: Joinpoint Regression

Joinpoint regression is implemented using the *segmented* package [21]. Four selection methods are available: fixed (user-specified, 0-5 joinpoints), Bayesian information criterion (BIC), default and recommended, Akaike information criterion (AIC), and permutation test (Monte Carlo). The method is selected via a dropdown in the module interface. Three model families are supported: log/multiplicative, linear/additive, and square root. For each segment, the application reports the annual percentage change (APC), its 95% CI, a 2-tailed *t* test, *P* value, and a significance flag. An overall average APC (AAPC) is computed as the segment-length-weighted mean of segment-specific APCs [5].

#### Module 5: Decomposition

Two decomposition methods are applied automatically based on data availability. When both number and rate metrics are present, Kitagawa 2-component decomposition [29] partitions the change in total case counts between 2 time points into a population-growth effect (mean rate multiplied by change in population) and an epidemiological effect (change in rate multiplied by mean population), using symmetric mid-point averages to ensure exact additivity. The 2 components sum to the observed change as an algebraic identity, holding to machine precision. When age-stratified rate rows are additionally present, Das Gupta 3-component decomposition [30] augments this with a population-aging component, applying proportional rescaling to preserve exact additivity. Both methods produce country-level waterfall charts showing the sign and magnitude of each component.

#### Module 6: Multimodel Time-Series Forecasting

Prior to model fitting, observations with missing or nonpositive rate values are excluded, because the Box-Cox log transformation is undefined at zero. This filter is applied consistently across the forecasting, EAPC, and joinpoint modules. Users analyzing rare diseases or low-count settings should be aware that this step may remove valid zero-count observations, and that the time-series index is built from the retained years in sequence, so any internal temporal gaps are treated as consecutive and are not interpolated. Three models are fitted for ensemble averaging: autoregressive integrated moving average (ARIMA; auto.arima), exponential smoothing state-space (EST, est), and a feed-forward neural network (nnetar) with a fixed random seed for reproducibility. Three additional models are computed and displayed as individual comparators but are not included in the ensemble: TBATS (tbats), Theta (thetaf), and Holt double-exponential smoothing (holt). The Ensemble model combines the ARIMA, ETS, and NNETAR forecasts using weights inversely proportional to their root-mean-square error (RMSE) in a 20% holdout, averaging both the point forecasts and the 80% and 95% prediction intervals. Equal weights are applied as a fallback when the series is too short for holdout evaluation or when a candidate model fails to converge. All 3 ensemble models, ARIMA, ETS, and NNETAR, apply Box-Cox log transformation with lambda=0. Bias-corrected back-transformation is enabled for ARIMA; ETS and NNETAR use the default back-transformation without bias adjustment. Among the comparators, Holt applies the same log transformation with bias-corrected back-transformation, Theta is fitted on the untransformed scale, and TBATS is fitted without an explicit transformation argument, so that any Box-Cox transformation is selected internally by the tbats implementation. An explicit nonnegativity floor, pmax(0, .), is applied to the point forecast and to all four prediction-interval bounds of every model, so that nonnegativity does not rely on the transformation alone. The ensemble output is floored in the same way and is in addition intrinsically nonnegative because it averages only log-transformed model outputs [6]. The forecast horizon is user-selectable from 5 to 30 years. Both 80% and 95% prediction intervals are displayed. Out-of-sample predictive accuracy of the candidate models is assessed by a 20% holdout cross-validation reporting RMSE, mean absolute error (MAE), and mean absolute percentage error (MAPE). Very short series or series containing abrupt structural breaks should be forecast and interpreted with particular caution.

#### Module 7: Descriptive Statistics

Seven commonly used exploratory chart types support exploratory analysis: time-trend lines, heatmaps, boxplots, sex comparison scatters, butterfly charts, 3D scatter, and treemaps.

#### Module 8: Health Frontier Analysis

The SDI-based health frontier is estimated by fitting a loess regression (span=0.8) of age-standardized mortality rate on SDI for all countries, using the most recent available SDI year per country [31]. The empirical frontier is defined as the loess-fitted value offset by a user-specified lower percentile of residuals (default: 10th). Country efficiency is reported as the ratio of frontier value to observed rate, expressed as a percentage. The loess-percentile frontier is intended as a descriptive, exploratory benchmark rather than a formal production-function estimate. Parametric methods such as data envelopment analysis and stochastic frontier analysis impose structural assumptions (eg, convexity or a specified inefficiency distribution) that are difficult to justify for cross-national burden data. The term efficiency here denotes relative position against the empirical frontier, not a normative or causal judgment. The smoothing span and residual percentile are user-adjustable, and analysts requiring formal efficiency scores should apply dedicated packages to the exported data.

#### Module 9: Descriptive APC Visualization

This module provides descriptive visualizations only and does not implement a formally identified APC framework. Marginal incidence rate ratios (IRR) are computed as the mean age-, period-, or cohort-specific rate divided by the overall mean. The period net drift, the one formally identified APC parameter [32], is estimated as 100 x (exp(beta) - 1) from a linear regression of log(rate) on period. Age and cohort marginal ratios are descriptive visualizations and must not be interpreted as independent or causal APC effects. Users requiring formal APC identification are directed to the NCI APC Web Tool or the *apc* *R package*.

#### Module 10: Health Inequality (Slope Index of Inequality and Relative Index of Inequality)

The slope index of inequality (SII) is estimated annually by assigning each country a fractional rank based on SDI and regressing the age-standardized rate on that rank using ordinary least squares. Population weighting by country size is identified as a planned methodological enhancement and is not applied in the current version. The relative index of inequality (RII) follows the Kunst-Mackenbach formulation [33] and is computed as the ratio of the model-predicted rate at the most disadvantaged (lowest-SDI) rank to that at the most advantaged (highest-SDI) rank, yielding a positive relative measure in which values above 1 indicate a greater burden among lower-SDI populations. Both indices are plotted over the selected period.

#### Module 11: Batch Export

All tabular results can be exported as a multisheet Excel workbook using the *openxlsx* package. Figures are exported as high-resolution PNG or PDF files via *ggplot2*. A structured methods-text paragraph, which carries a disclaimer reminding users to review and adapt it to their specific analysis before any publication use, is auto-generated in plain text and Word-compatible format (*officer*).

### Internal Consistency Safeguards

Three design decisions enforce internal consistency of output by construction. First, Kitagawa additivity: the symmetric mid-point formulation ensures that population-growth and epidemiological components sum exactly to the observed change in counts as an algebraic identity, holding to machine precision. A PASS or FAIL additivity check is reported alongside every decomposition output. Second, forecast nonnegativity: the 3 ensemble models (ARIMA, ETS, and NNETAR) apply Box-Cox log transformation with lambda=0, and every model, including the comparators, applies an explicit nonnegativity floor to its point forecast and to both prediction intervals, so that no negative rate can be reported. Third, frontier input restriction: the frontier module silently restricts input to rate-metric, age-standardized rows, preventing accidental frontier estimation on mixed metrics. Additional robustness features include reproducible neural-network forecasts via a fixed random seed, explicit user control over joinpoint count, and automatic dataset auditing on upload.

### Licensing, Source Code, and Deployment

The GBD Analysis Suite is released under the MIT open-source license. Source code, a bundled 196-country demonstration dataset (1990 to 2023), and a one-line package-installation script are publicly available at the GitHub repository [27]. The deployed application is accessible at the Shiny app [26]. A complete sessionInfo() output from the deployed environment is provided in Multimedia Appendix 1.

### Ethical Considerations

This paper describes an open-source software tool and does not involve primary human participant research. The dashboard operates exclusively on publicly available, de-identified, population-level aggregate outputs from the GBD study. (1) Formal ethics review was not required because no human participants were enrolled, no patient data were accessed, and no individual-level information is handled at any stage. (2) Informed consent is not applicable. (3) No personal or identifiable data are processed. (4) No participants were involved. Compensation is not applicable. (5) All figures depict only aggregate population-level data. No identifiable individuals appear. Users retain full ownership of any data they upload. The deployed instance processes uploaded data only in active-session memory and does not persist user-uploaded files beyond session termination.

## Results

### Application Availability and Deployment

The GBD Analysis Suite is deployed and publicly accessible at the Shiny app [26]. Source code, the bundled 196-country demonstration dataset, and installation instructions are available at the GitHub repository [27] under the MIT license. The application loads and executes in any modern web browser (Chrome, Firefox, Safari, and Edge) without requiring a local R installation, Java runtime, or institutional software license.

### Module Coverage

Table 1 summarizes the 11 modules, the primary *R package* each relies upon, the required input columns, and the outputs produced. Every module produces at least one tabular output and at least one figure, all of which are included in batch exports.

**Table 1:** Analytical modules of the GBD Analysis Suite.

	Module	Analytical use^a^	Main statistical procedure	Primary R packages	Key references
1	Data import and validation	Upload, auto-detect columns, validate structure, and audit dataset composition	Column alias detection, structured data-quality tabulation by measure, metric, sex, and age	*readxl, readr*	None
2	Choropleth maps	Map age-standardized rates, EAPC^b^ magnitude, or percentage change across countries	Name-matched polygon join to a standardized world layer, sequential/diverging color scaling	*maps, plotly*	None
3	EAPC	Quantify and classify the average annual trend in age-standardized rates	Log-linear regression of ln(rate) on year, EAPC = 100 × (exp(beta) – 1) with 95% CI	*stats (lm)*	[28]
4	Joinpoint regression	Detect changepoints and estimate segment-specific trends	Segmented (broken-line) regression, segment APC^c^ and length-weighted AAPC^d^, fixed, BIC^e^, AIC^f^, or permutation selection	*segmented*	[5,21]
5	Decomposition	Partition the change in total counts into population and epidemiological components	Kitagawa 2-component and Das Gupta 3-component decomposition with mid-point/proportional balancing	*dplyr*	[29,30]
6	Multimodel time-series forecasting	Project future rates with prediction intervals and compare candidate models	ARIMA^g^/ETS/NNETAR inverse-RMSE weighted ensemble plus TBATS, Theta, and Holt comparators, Box-Cox (lambda = 0) on ensemble members and Holt, nonnegativity floor on all models, 20% holdout RMSE^h^/MAE^i^/MAPE^j^	*forecast*	[6]
7	Descriptive statistics	Provide exploratory visual summaries across years, sexes, ages, and locations	Seven exploratory chart types, including trend lines, heatmaps, boxplots, scatters, and treemaps	*ggplot2, plotly*	None
8	Health frontier analysis	Benchmark country rates against an empirical SDI^k^-based frontier	Locally weighted regression (LOESS, span 0.8) shifted by a user-specified residual percentile	*stats (loess)*	[31]
9	Descriptive APC visualization	Display descriptive age, period, and cohort patterns and the period net drift	Marginal incidence-rate ratios (descriptive), period net drift = 100 × (exp(beta) – 1) from log(rate) on period	*ggplot2*	[32]
10	Health inequality (SII^l^ and RII^m^)	Summarize the cross-national socioeconomic gradient in rates	Slope and relative index of inequality from fractional-rank regression (unweighted OLS)	*stats (lm)*	[33]
11	Batch export	Export all tabular and figure outputs plus an auto-generated methods paragraph	Multisheet workbook writing, high-resolution figure rendering, templated methods-text generation	*openxlsx, officer*	None

^a^Analytical use summarizes each module's intended descriptive or analytical purpose. The main statistical procedure lists the principal computation performed. References indicate the key methodological source for the procedure, where applicable. Modules built primarily on general-purpose visualization or data-handling packages are listed as “None.” All modules operate on the core input fields (location, year, and value) and use additional fields where available.

^b^EAPC: estimated annual percentage change.

^c^APC: annual percentage change.

^d^AAPC: average annual percentage change.

^e^BIC: Bayesian information criterion.

^f^AIC: Akaike information criterion.

^g^ARIMA: autoregressive integrated moving average.

^h^RMSE: root mean square error.

^i^MAE: mean absolute error.

^j^MAPE: mean absolute percentage error.

^k^SDI: sociodemographic index.

^l^SII: slope index of inequality.

^m^RII: relative index of inequality.

### Feature Comparison With Existing Tools

Table 2 compares the GBD Analysis Suite against 5 established tools covering parts of the GBD analytical workflow. Across 14 capability dimensions spanning data input, the 6 core analytical methods of modern GBD papers, and output and deployment features, the GBD Analysis Suite is the only platform supporting all analytical dimensions without requiring programming expertise. Two rows at the bottom of the table, peer-reviewed methodology validation and maturity of established user base, note dimensions on which competing tools currently have an advantage over this newly released suite. These comparisons should be read in context: the comparator tools differ in primary intent (eg, GBD Compare is an IHME visualization portal of official estimates; NCI Joinpoint is a single-method analytical program). The suite's contribution is integration breadth, not replacement of any individual specialist tool. Comparator tools were selected against three criteria: publicly available or academically published tools designed for population-level epidemiological data, tools capable of accepting user-uploaded GBD-format input files, and tools addressing at least two of the analytical domains covered by the suite. This comparison is not exhaustive, and specialized packages exist for individual methods.

**Table 2:** Feature comparison across six burden of disease analysis platforms.

Capability	This suite	GBD^a^ compare	GBD foresight	NCI^b^ joinpoint	BAPC (R)	NordPred (R)
User-uploaded data accepted	Yes	No	No	Yes	Yes	Yes
Code-free interface	Yes	Yes	Yes	Yes	No	No
Open source (code available)	Yes	No	No	Partial^c^	Yes	Yes
Browser-deployable	Yes	Yes	Yes	No	No	No
Runs without local R install	Yes^d^	Yes	Yes	Partial^e^	No	No
EAPC computation	Yes	No	No	No	No	No
Joinpoint regression	Yes	No	No	Yes	No	No
Multimodel forecasting	Yes^f^	No	Partial^g^	No	Partial^h^	Partial^h^
Kitagawa decomposition	Yes	No	No	No	No	No
Das Gupta decomposition	Yes^i^	No	No	No	No	No
SII^j^ and RII^k^ inequality	Yes	No	No	No	No	No
SDI^l^-based frontier analysis	Yes	No	No	No	No	No
APC^m^ visualization with net drift	Yes	No	No	No	Yes	Partial^n^
Batch export (Excel, figures, methods text)	Yes	Partial^o^	Partial^o^	Yes	No	No
Peer-reviewed methodology validation	No^p^	Yes	Yes	Yes	Yes	Yes
Maturity/established user base	New^q^	Established	Established	Established	Established	Established

^a^GBD: Global Burden of Disease.

^b^NCI: National Cancer Institute.

^c^NCI Joinpoint binary is freely distributed, but the source is not public.

^d^Accessible via shinyapps.io in any modern browser.

^e^Windows desktop client only.

^f^Three-model inverse root mean square error–weighted ensemble (autoregressive integrated moving average [ARIMA], ETS, NNETAR) plus three individual comparators (TBATS, Theta, Holt).

^g^GBD Foresight provides Institute for Health Metrics and Evaluation (IHME)–produced forecasts only. No user-configurable models.

^h^BAPC and NordPred focus on Bayesian APC forecasting for cancer incidence. Single-model approach.

^i^Activates automatically when age-stratified number and rate data are provided.

^j^SII: slope index of inequality.

^k^RII: relative index of inequality.

^l^SDI: sociodemographic index.

^m^APC: annual percentage change.

^n^NordPred produces APC-structured forecasts but does not offer separate APC visualization.

^o^Image export only. No multisheet workbook or structured methods text.

^p^This manuscript constitutes the first formal description. Methodology not previously peer-reviewed.

^q^New release. User base is limited at time of publication.

### Performance Characteristics

Runtime performance was measured on the bundled 196-country, 34-year demonstration dataset (599,760 observations of GBD 2023 meningitis estimates) on a four-core, eight-thread workstation with 30 GB of RAM running R 4.5.3 under Windows 10. Interactive modules, including data import validation, descriptive visualization, EAPC estimation, joinpoint regression, and inequality computation, completed in under 2 seconds per user action, with a measured range of 0.1 to 1.9 seconds. Forecasting was the most computationally intensive module, with the 3-model ensemble (ARIMA, ETS, and NNETAR) plus 3 individual comparators (TBATS, Theta, and Holt), requiring approximately 9 seconds per country at a 15-year horizon. Exporting the complete set of outputs took approximately 12 seconds in total, comprising approximately 1 second for a 10-sheet Excel workbook, whose sheets are written according to which analyses have been run, approximately 10 seconds for the high-resolution figures, which are saved individually at 300 dpi in PNG or PDF format, and under 0.1 seconds for the auto-generated methods text file. Peak memory footprint during full-dataset operations was approximately 600 MB. The free shinyapps.io tier did not complete loading the full demonstration dataset within 60 seconds in our testing, so users working with full multicountry datasets are advised to run the application locally via shiny::runApp() on a machine with at least 4 GB of available memory rather than relying on the shared public instance. Smaller datasets (single-country or single-cause extracts, typically 1000 to 10,000 observations) executed all modules in under 1 second sequentially, at the memory footprints reported in Table 3. The application has been exercised with input datasets up to approximately 1 million rows without structural failure, completing descriptive and EAPC computation on 1 million rows in approximately 5 seconds at a peak footprint of approximately 800 MB. Response times scale approximately linearly with row count.

**Table 3:** Performance benchmark of the analytical engines across input sizes (median of three runs).

Rows^a^	Countries	File size (MB)	Load + parse (s)	Analysis (s)	Peak memory (MB)
1000	1	0.15	0	0.11	252.7
5000	2	0.72	0	0.12	253.2
10,000	4	1.38	0.01	0.61	341.0
50,000	17	6.43	0.04	0.86	415.2
100,000	33	12.66	0.07	1.19	434.2
599,760	196	88.63	0.39	5.94	582.7

^a^Subsets were genuine head-N samples of the demonstration dataset written to disk as CSV. Load + parse covers data.table reading plus the application column parser. Analysis covers the chained descriptive, estimated annual percentage change, inequality, slope index of inequality (SII), and frontier engines. Peak memory is the maximum R memory observed during load and analysis. The 1000- and 5000-row tiers contain only one to two countries, so the inequality, SII, and frontier engines (which require three or more countries) do not execute at those sizes.

To characterize this scaling formally, the application's analytical engines were benchmarked on genuine subsets of the demonstration dataset ranging from 1000 to 599,760 rows. Table 3 reports the median of 3 runs per size tier. This benchmark times data loading and parsing plus the core analytical pipeline (descriptive statistics, EAPC, health inequality, SII, and frontier computation). It does not include the heavier figure-rendering and workbook-writing steps of a full batch export, which account for the longer end-to-end export time noted above. Load and parse time scaled approximately linearly with file size, while analytical time scaled sub-linearly with row count and approximately linearly with the number of countries, because the inequality, SII, and frontier engines operate on per-country and per-year aggregates rather than on raw row count. The complete 599,760-row file (88.6 MB across 196 countries) was processed by the analytical pipeline in approximately 5.9 seconds at a peak memory footprint of approximately 583 MB.

### Demonstration Outputs

**Figure 1:**
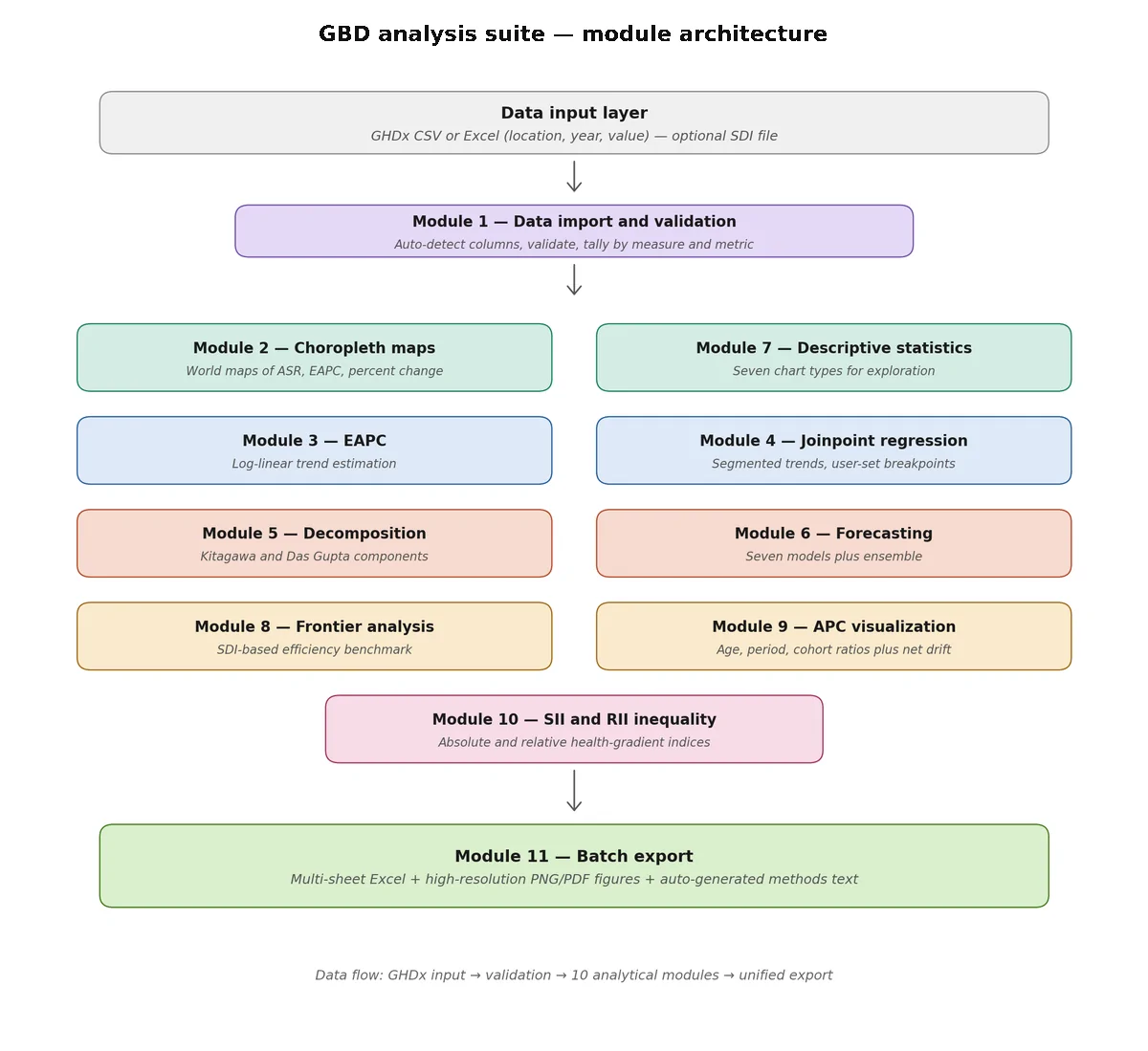
Module architecture of the GBD Analysis Suite, showing the data input layer, 10 analytical modules organized by function, and the unified batch-export module. APC: annual percentage change; ASR: age-standardized rate; EAPC: estimated annual percentage change; GBD: global burden of disease; GHDx: Global Health Data Exchange; RII: relative index of inequality; SDI: sociodemographic index; SII: slope index of inequality.

**Figure 2:**
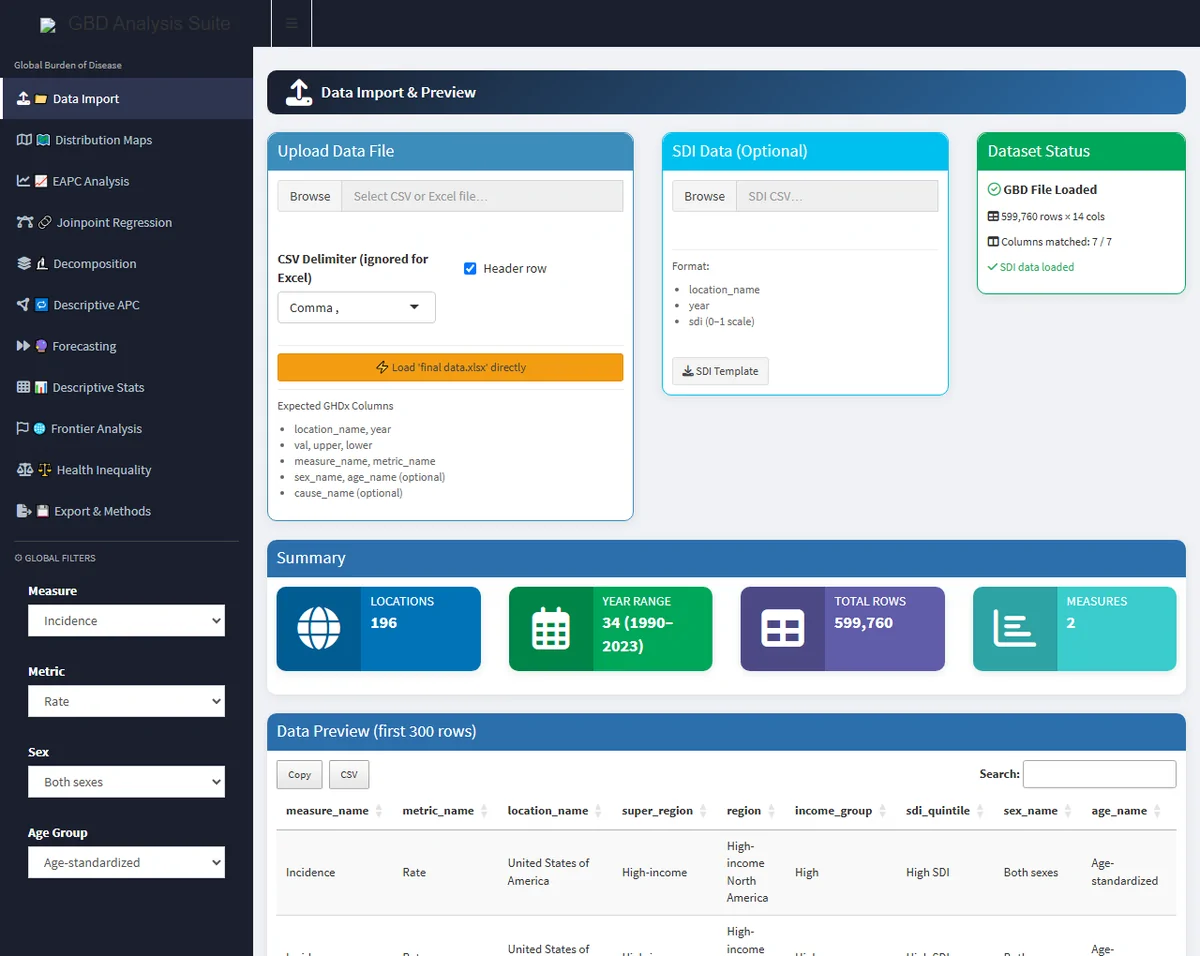
Dashboard interface of the GBD Analysis Suite, data import view, showing the 11-tab sidebar navigation, the module workspace, the data quality report, and the data preview for the bundled 196-country demonstration dataset. APC: annual percentage change; EAPC: estimated annual percentage change; GBD: global burden of disease; SDI: sociodemographic index.

**Figure 3:**
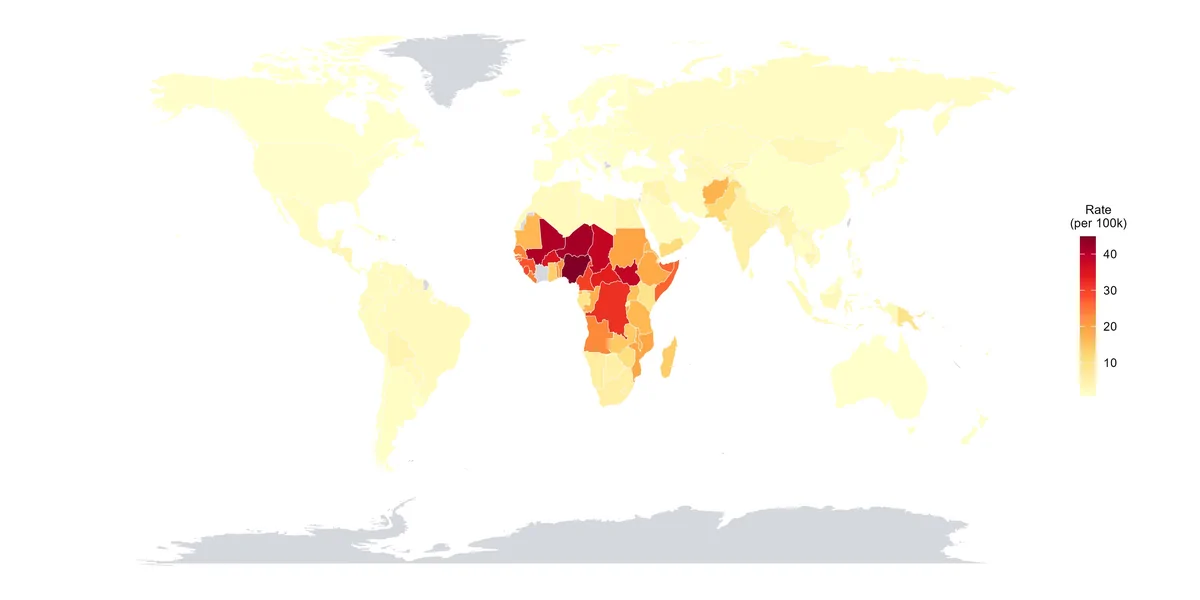
Global choropleth map of age-standardized rates in 2023 across 196 countries and territories (bundled demonstration dataset).

**Figure 4:**
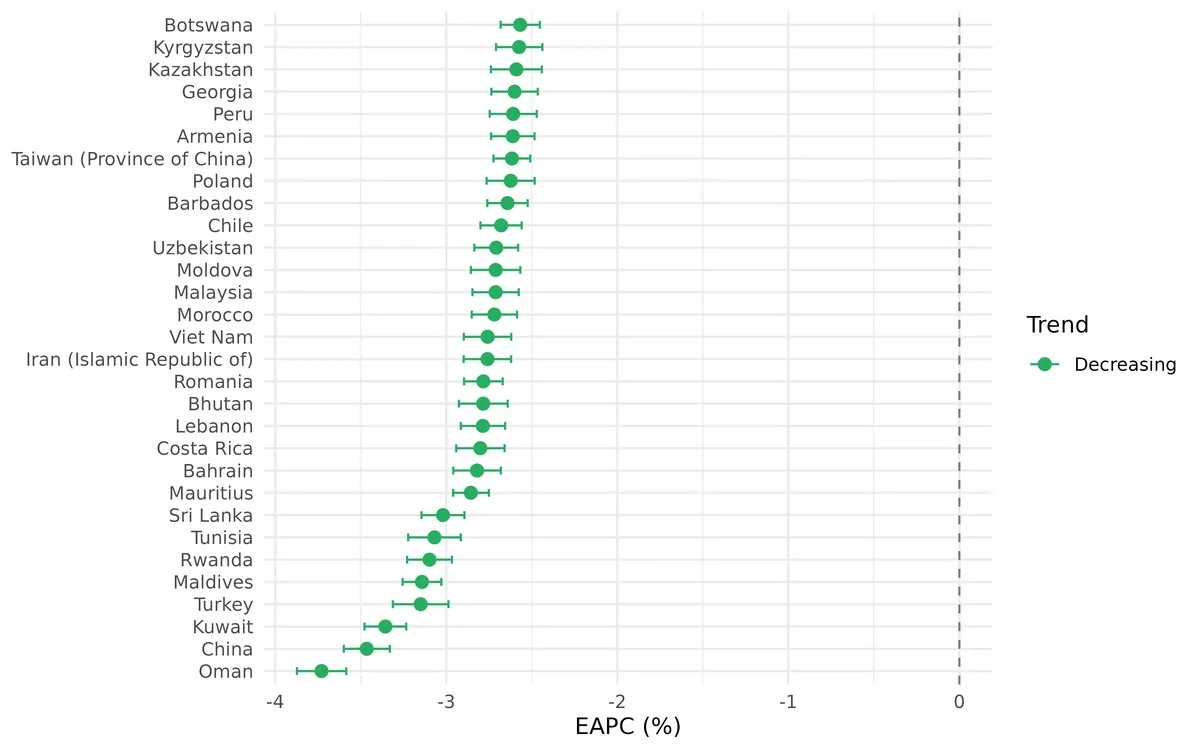
Forest plot of estimated annual percentage change (EAPC) in age-standardized rates across 30 selected countries, 1990 to 2023.

**Figure 5:**
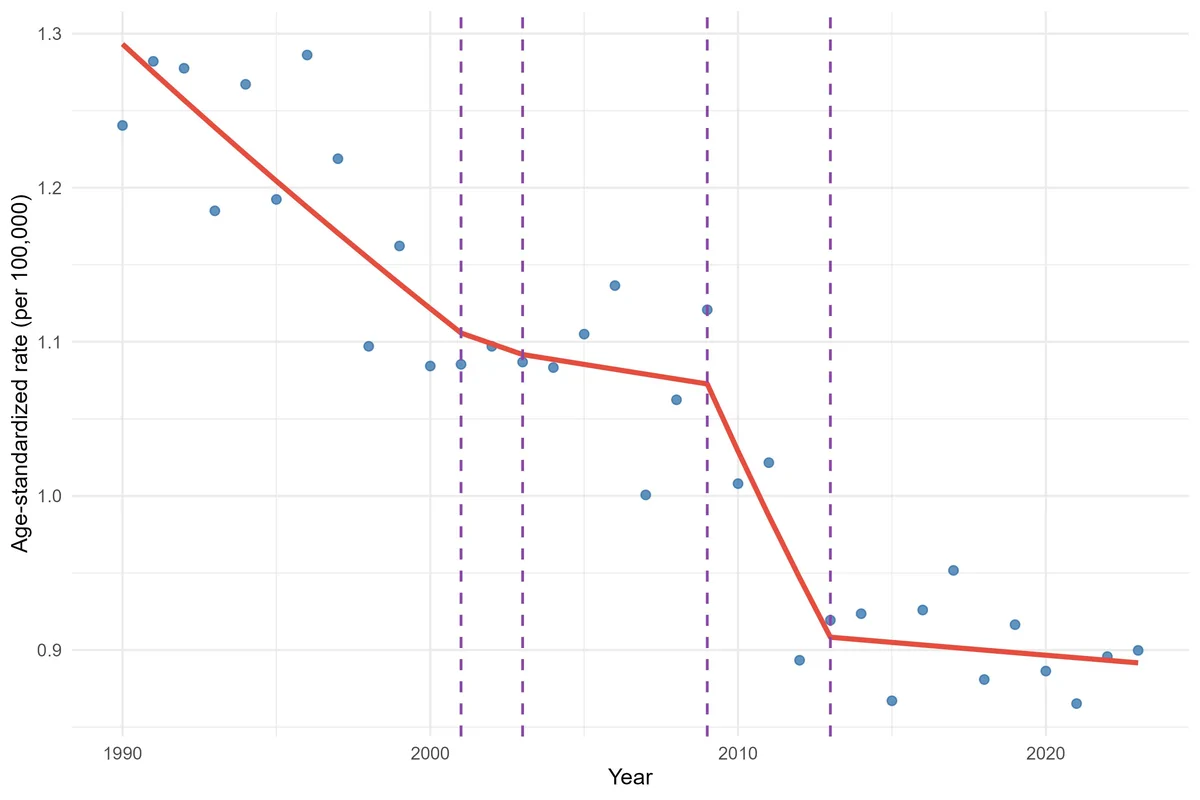
Joinpoint regression output for a representative demonstration country, fitted with 4 user-specified joinpoints.

**Figure 6:**
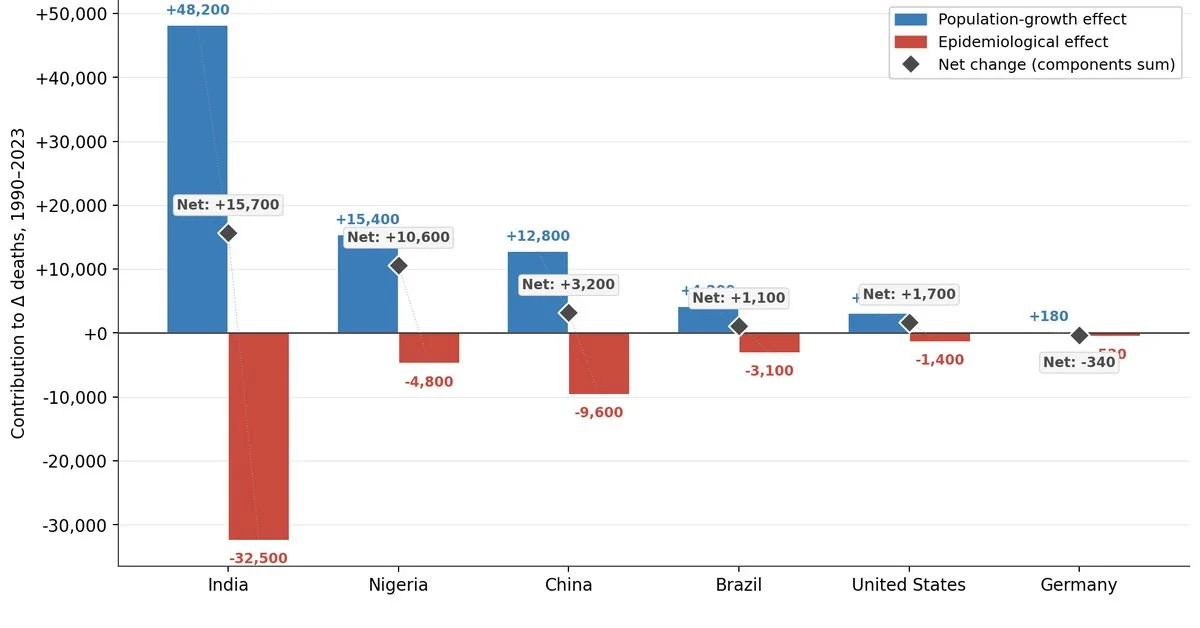
Kitagawa decomposition of change in total deaths across six representative countries, 1990 to 2023, showing the population-growth and epidemiological components.

**Figure 7:**
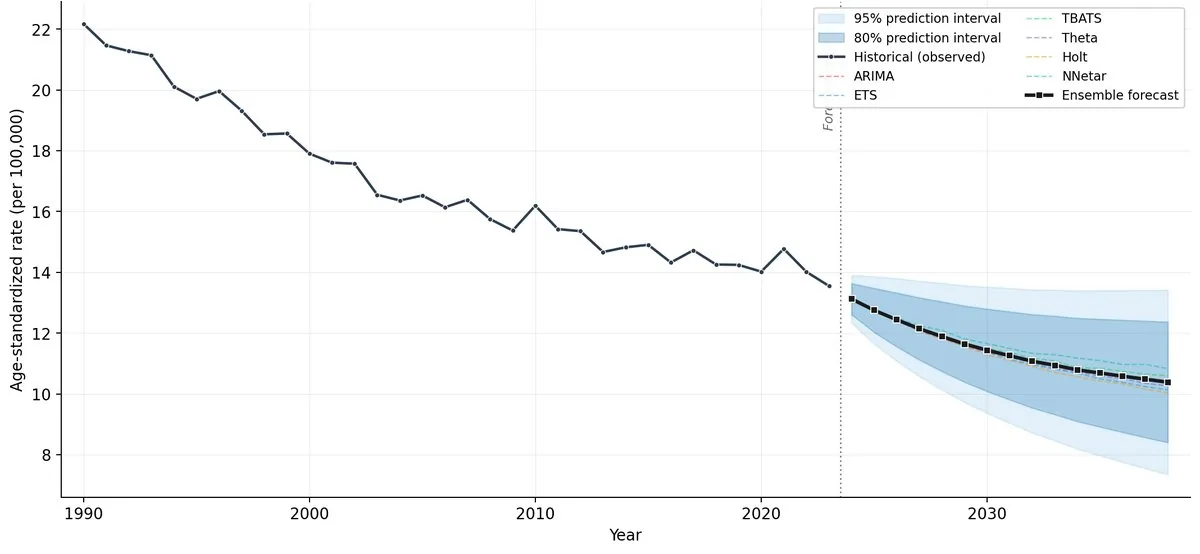
Multimodel time-series forecast of age-standardized rates (2024 to 2038) from the three-model ensemble (autoregressive integrated moving average, ETS, and NNETAR) with comparators, with 80% and 95% prediction intervals.

**Figure 8:**
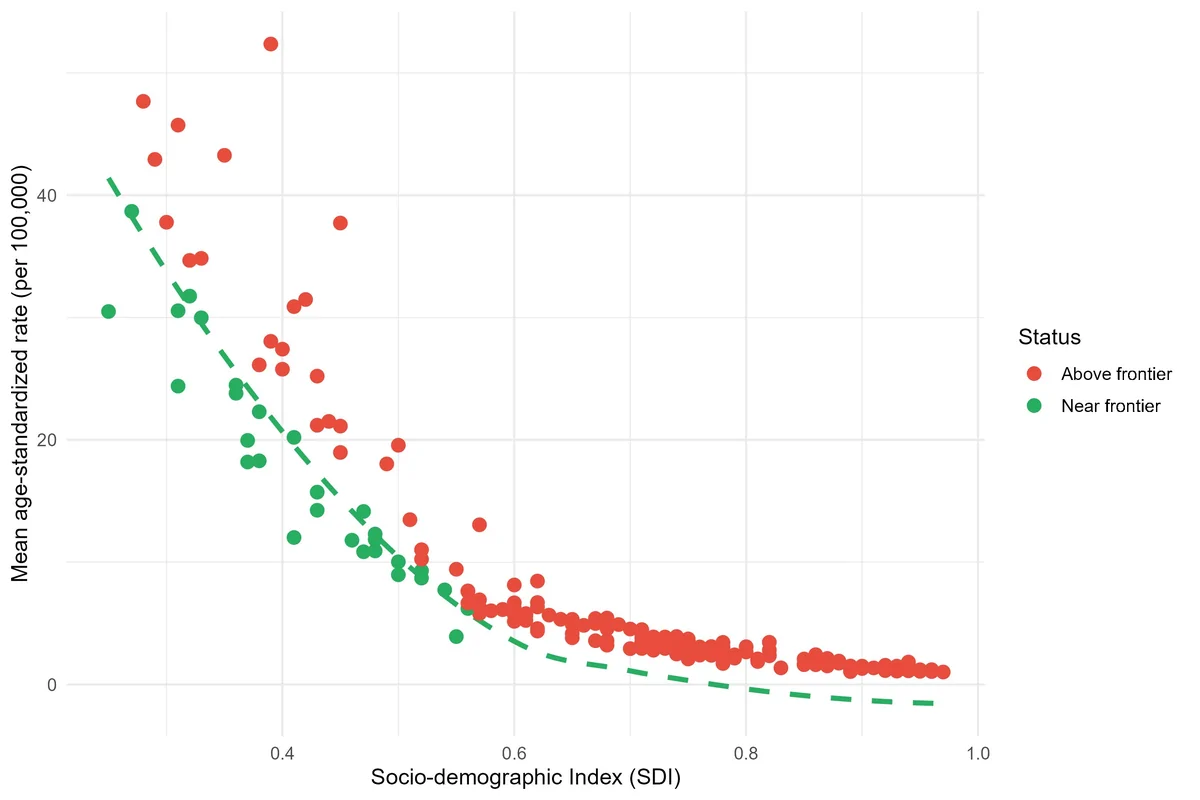
Sociodemographic index (SDI)–based health frontier analysis across 196 countries in the most recent year, with near-frontier and above-frontier country classification.

**Figure 9:**
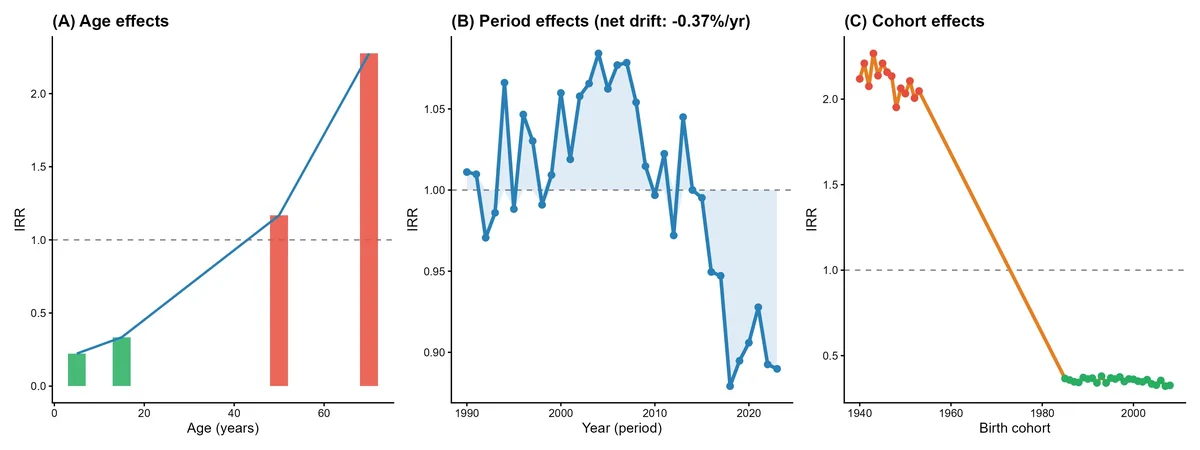
Descriptive annual percentage change visualization for a demonstration country, showing age effects, period effects with net drift, and cohort effects. IRR: incidence rate ratio.

**Figure 10:**
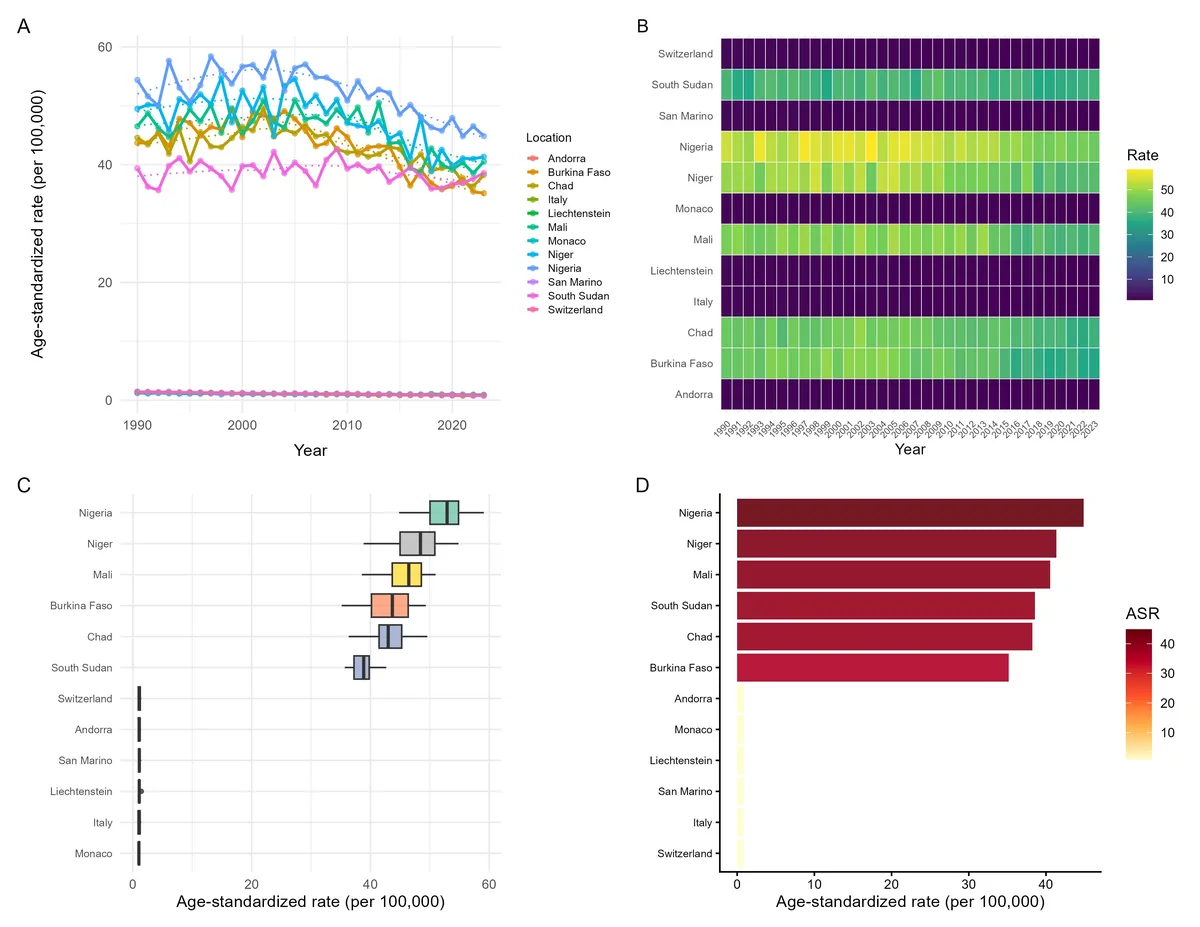
Module 7 descriptive statistics panel for the bundled demonstration dataset, illustrating four of the module's exploratory chart types: temporal trend lines across selected countries, a country-by-year rate heatmap, rate-distribution boxplots, and an age-standardized burden ranking for the most recent year. ASR: age-standardized rate.

**Figure 11:**
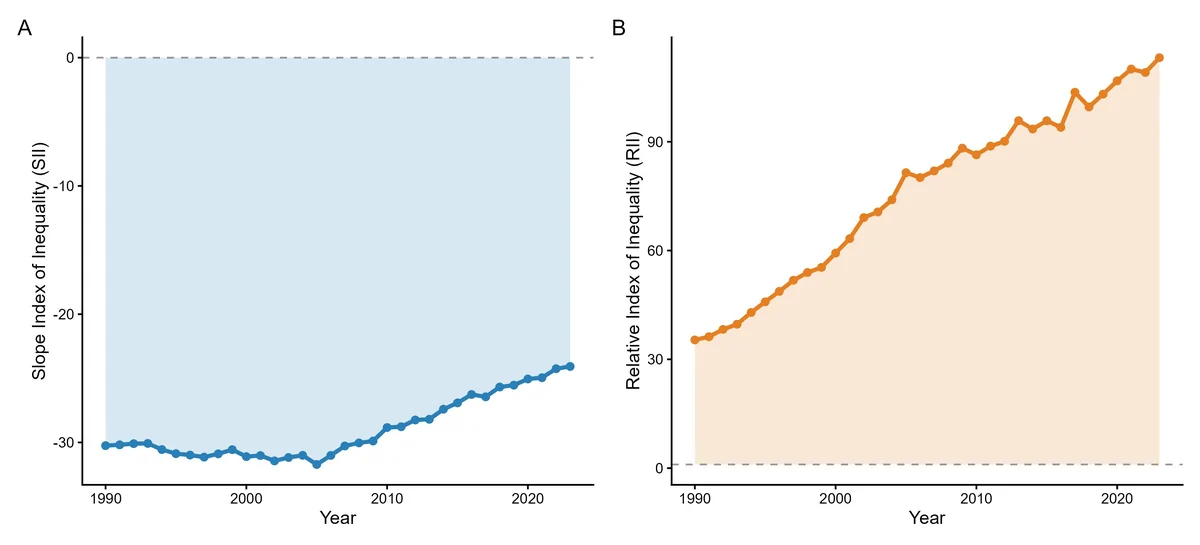
Health inequality output for the bundled demonstration dataset, showing the (A) SII and (B) RII over time, with countries ranked by sociodemographic index. Negative slope index of inequality values indicate that burden is concentrated in lower-sociodemographic index countries.

The bundled 196-country demonstration dataset allows new users to execute the full 11-module workflow with a single button click, producing all tabular and figure outputs within one session. Figure 1 presents the application’s module architecture. Figure 2 shows the main dashboard interface. Figures 3-9 present representative outputs from the choropleth mapping (Figure 3), EAPC estimation (Figure 4), joinpoint regression (Figure 5), Kitagawa decomposition (Figure 6), ensemble forecasting (Figure 7), frontier analysis (Figure 8), and descriptive APC (Figure 9) modules, all computed on the demonstration dataset. Figure 10 presents the descriptive statistics panel (Module 7) and Figure 11 the health inequality output (Module 10), both computed on the same dataset.

The descriptive statistics module (Module 7) generates a panel of commonly used exploration chart types from a single uploaded dataset. Figure 10 illustrates four of these for the bundled demonstration dataset: temporal trend lines of age-standardized rates across selected countries, a country-by-year rate heatmap that summarizes the full panel at a glance, rate-distribution boxplots that compare the spread of values across locations, and an age-standardized burden ranking bar chart for the most recent year. The module additionally offers sex comparison scatters, a 3D scatter, and a treemap, giving seven exploratory chart types in total. Each chart is rendered interactively and can be exported as a high-resolution image. The panel is intended for rapid visual screening of a dataset before formal analysis, and the figures shown here required no programming beyond uploading the data file.

The Health Inequality module (Module 10) quantifies the cross-national socioeconomic gradient in burden using the SII and the RII, derived from regression of age-standardized rates on the fractional rank of the SDI across all available countries. Figure 11 shows both indices over the full 1990 to 2023 period for the demonstration dataset. The SII is negative throughout, reflecting that the highest burden is carried by the lowest-SDI countries, and its magnitude narrows over time, consistent with a gradually attenuating absolute gradient. Both indices are computed with unweighted ordinary least squares as described in the Methods. Population-weighted estimation is identified as a planned enhancement.

### Extensibility

The module-level architecture is designed to be flexible across cause, country set, and time window, subject to input data compatibility. Each module operates on the subset of uploaded data matching its required columns, with no hard-coded assumptions about which countries, years, or causes are present. The application has been exercised on single-cause datasets (cancer incidence, cardiovascular mortality, and infectious disease burden), multicause noncommunicable disease extracts, single-country historical trend analyses, and multicountry regional comparisons, across time horizons ranging from 10 to 34 years. New analytical methods can be added as additional Shiny tabs following the existing module pattern. Contributions via the public GitHub repository are welcomed.

## Discussion

### Principal Contribution

This paper describes the GBD Analysis Suite, an open-source R Shiny dashboard that integrates 11 commonly used analytical modules for GBD-style burden of disease research into a single, code-free web interface. The application fills a structural gap in the existing tool ecosystem: platforms optimized for exploration (GBD Compare [3], GBD Foresight [4]) do not support user-uploaded data or analytical recomputation, while platforms optimized for analysis (NCI Joinpoint [5], BAPC [7], NordPred [8]) are single-method tools requiring local installation and, in most cases, programming fluency. The GBD Analysis Suite occupies the unfilled niche of a browser-accessible, user-uploadable, multimethod analytical platform that supports the complete analytical workflow of a modern GBD paper.

### Design Principles

Three design principles guided development. First, accessibility: the application runs in any browser without a local R installation, commercial software license, or specialized infrastructure, removing barriers that disproportionately affect investigators in low- and middle-income countries. Second, reproducibility: the application enforces internal consistency by construction (Kitagawa additivity, forecast nonnegativity, frontier input validation), uses fixed random seeds for stochastic components, and exports a structured methods-text paragraph alongside numerical output. Third, transparency: all source code is public, all statistical procedures are documented with literature citations in both the application interface and the exported methods text, and every export includes software version and dependency information.

### Intended Use Cases

The application is designed for the analyst who has downloaded a GHDx cause-country-year extract and wants to produce export-formatted trend, decomposition, forecast, frontier, and inequality outputs without assembling and maintaining a bespoke R codebase. Typical workflows include cause-specific burden papers for single-country or multicountry analyses, regional or multicountry comparisons with within-region frontier and SII/RII computation, methodological teaching and training using the bundled demonstration dataset, and rapid secondary analysis as GHDx data are updated, supporting reproducible reruns across GBD releases. Conversely, the platform is intended for descriptive and exploratory analysis of population-level, aggregate GHDx-format data. It is not a substitute for primary statistical software where formal inferential guarantees are required, and it is not appropriate for individual-level data, small-area analyses, or studies requiring formal inference from an identified APC model. Users working with sparse data or non-GHDx datasets should treat outputs as preliminary.

### Implications for Low- and Middle-Income Country Research

A primary design motivation was to lower the technical barrier to GBD-style analyses for investigators in LMIC settings, where biostatistical support, commercial software licenses, and access to high-performance computing are often limited [11]. The application runs entirely in the browser, requires no local R installation, and accepts GHDx CSV files directly, the same freely distributed format used by every GBD publication. The batch-export feature produces an export-ready methods paragraph alongside numerical outputs, reducing the preparation burden for peer-reviewed submission. Given the dual constraint of high burden of disease needs and limited analytical infrastructure that characterizes much of the LMIC research environment, a browser-native, code-free platform addresses a practical gap that code-based tools do not fill.

### Limitations

Several limitations bear consideration. First, the application operates on GBD estimates, which themselves carry uncertainty from data sparsity, garbage-code redistribution, and the IHME modeling assumptions that generate them [1]. The application detects upper and lower uncertainty interval columns in uploaded data but does not propagate them through analyses. All computations use point estimates only. Second, EAPC is calculated from age-standardized rates using the IHME age-standard, which differs from the Segi world standard commonly used in cancer-registry analyses. Comparisons across the literature using different standards require care. Third, the APC module computes only descriptive marginal ratios plus the identified period net drift. Formal identifiable APC inference requires constraint-based estimation and is not provided. Users needing this are directed to dedicated packages. Fourth, forecast prediction intervals reflect parametric uncertainty within each model family and do not fully capture model-selection uncertainty beyond the ensemble averaging provided. Fifth, although the joinpoint module now provides automated BIC (default, recommended), AIC, and permutation-test selection methods, analyses requiring strict cross-study replicability should use the fixed mode with an explicitly stated breakpoint count, as BIC and permutation methods may yield different optimal breakpoints across software versions. Sixth, while the application has been exercised on a range of disease, country, and time-window configurations during development, formal performance benchmarking across the full space of possible GBD queries has not been conducted, and formal unit-test coverage with continuous integration is currently limited. Users are encouraged to verify outputs against known reference datasets before publication. Seventh, the application currently supports GHDx data exports only. Extensions to accept cancer-registry (IARC/CI5), vital registration (World Health Organization [WHO] mortality database), or other burden data sources would require additional column-mapping logic. Eighth, uploaded data in the deployed instance are held in session memory only and are not persisted beyond the active session, but users with formal data-governance requirements, for example those handling restricted-access extracts under institutional data-use agreements, should deploy the open-source code on local or institutional infrastructure rather than using the public shinyapps.io instance. Finally, long-term sustainability depends on continued maintenance by the project’s contributors. The MIT-licensed codebase and public GitHub repository allow for community forks or handover should active development pause.

### Strengths

Despite these limitations, the developed platform has several strengths. To our knowledge, it is among the first freely available, browser-accessible platforms to integrate the full multimethod workflow of a modern GBD burden of disease study in a code-free interface. The design emphasizes reproducibility through enforced internal consistency (Kitagawa additivity, forecast nonnegativity, frontier input restriction), fixed random seeds, and auto-generated methods text that accompanies every export. Deployment to shinyapps.io requires no institutional infrastructure, making the tool immediately accessible to researchers in LMIC settings where commercial software licenses and high-performance computing resources are often unavailable. All source code is released under the MIT license on a public GitHub repository, supporting community forks, contributions, and independent audit of methodology.

### Future Directions

Planned extensions include support for additional data sources beyond GHDx (particularly IARC CI5 cancer registry data and WHO Mortality Database exports), a comparison mode displaying side-by-side outputs from two uploaded datasets (for example, two GBD releases, or model versus registry data), integration with bibliographic tools (Zotero, Mendeley) to auto-import relevant citations into the exported methods text, a server-side caching layer for faster response on large multicountry datasets, and progressive expansion of unit-test coverage toward a continuous-integration pipeline. Contributions from the community are welcomed via the public GitHub repository.

### Conclusions

The GBD Analysis Suite integrates 11 commonly used analytical modules for GBD-style burden of disease research into a single, open-source, code-free web interface. The platform fills a structural gap between exploration-only visualization portals and single-method analytical packages and is freely deployable without institutional infrastructure. By lowering the technical barrier to a reproducible, multimethod analytical workflow, the application aims to expand the community of researchers able to conduct structured, reproducible GBD-style analyses. Translating these outputs into peer-reviewed evidence requires appropriate epidemiological expertise and critical interpretation.

## Appendix

Multimedia Appendix 1 sessionInfo() output from the deployed environment.

## Abbreviations

AAPC: average annual percentage change
AIC: Akaike information criterion
APC: annual percentage change
APF: article processing fee
ARIMA: autoregressive integrated moving average
ASR: age-standardized rate
BAPC: Bayesian age-period-cohort
BIC: Bayesian information criterion
EAPC: estimated annual percentage change
ETS: exponential smoothing state-space
GBD: Global Burden of Disease
GHDx: Global Health Data Exchange
IARC: International Agency for Research on Cancer
IHME: Institute for Health Metrics and Evaluation
IRR: incidence rate ratio
LMICs: low and middle-income countries
LOESS: locally estimated scatterplot smoothing
MAE: mean absolute error
MAPE: mean absolute percentage error
NCI: National Cancer Institute
NNETAR: neural network autoregression
RII: relative index of inequality
RMSE: root mean square error
SDI: sociodemographic index
SII: slope index of inequality
TBATS: trigonometric seasonality, Box-Cox transformation, ARMA errors, trend and seasonal components
WHO: World Health Organization

## Data Availability

This paper describes software methodology. No new empirical data were generated. The application source code and bundled demonstration dataset are publicly available at GitHub repository [27] under the MIT license. The deployed application is accessible at the Shiny app [26]. A complete sessionInfo() output is provided in Multimedia Appendix 1. Underlying GBD data are available through the Global Health Data Exchange (GHDx) [12].
